# Conserving diggers: from gold miners to aardvarks

**DOI:** 10.1093/conphys/coy024

**Published:** 2018-05-08

**Authors:** Duncan Mitchell

**Affiliations:** 1 Brain Function Research Group, School of Physiology, Faculty of Health Sciences, University of the Witwatersrand, Johannesburg, South Africa; 2 School of Human Sciences, Faculty of Science, University of Western Australia, Perth, Australia

From developing heat stress assessments to keep miners safe 3 km underground in the hot and humid stopes of South African gold mines (Mitchell and Whillier, 1971) to understanding aardvark energetic needs in the Kalahari Desert; that is how my research career has wandered. I can draw its roadmap but only retrospectively, and I suspect that I am not alone among researchers in relying on a retrospective roadmap. I came into thermal physiology from an education in experimental physics. My physics professor told me that I would never be any good in physics; ‘just useful’. The background in physics did mean that I was comfortable with heat transfer equations but that was not its most-important legacy for my career in conservation physiology. Experimental physics taught me how to make measurements. As an Honours (fourth-year) student I had to measure the charge on the electron using the oil drop method that got Millikan a Nobel Prize.

My contribution to conservation physiology, I hope, has been measurements of physiological function of individual wild animals living free in their natural habitats. Conservation-related measurements of individual physiological function are uncommon for any vertebrate, and especially for mammals; for vertebrates living free in their natural habitats they are rare. While laboratory experiments have delivered crucial knowledge on what vertebrates can achieve physiologically, only studies of free-living vertebrates in their natural habitats, where individuals are subject to complex stressors, tell us what animals actually will do. It is also important to make physiological measurements with humans around as little as possible because wild vertebrates, perhaps with the exceptions of habituated primates and cetaceans, are afraid of humans. So the physiology, including both the behaviour and the autonomic function, that wild vertebrates display in the presence of human observers is not normal physiology—it’s physiology clouded by fear.

**Figure 1:**
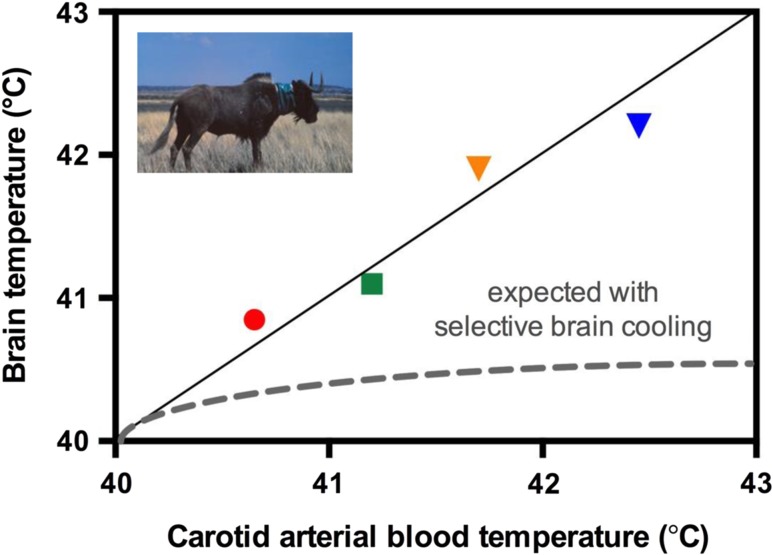
Maximum brain (hypothalamic) and carotid artery blood temperatures, measured by biologging, in four black wildebeest *Connochaetes gnou* fleeing to avoid capture from a helicopter, together with brain temperatures expected from selective brain cooling (Jessen *et al.*, 1994). Solid line is the line of identity.

Fear contributed to what I believe are the four most-important data points of my research career (Fig. 1). With the late Claus Jessen (Justus Liebig University, Germany), whom I and many others regard as the pre-eminent thermal physiologist of the late 20th century, and a team of colleagues, I had been measuring brain and body temperatures in free-living black wildebeest *Connochaetes gnou*, using biologgers attached to the animals. To recover our biologgers, we needed to recapture the animals from the South African savannah, a process for which the wildebeest were not willing to volunteer. Fortunately for us, the veterinary and pilot staff of South African National Parks are expert in capturing large mammals by chemical immobilization (‘darting’) from helicopters. That required a chase, with the wildebeest fleeing from the helicopter, as if from a lion. When we downloaded the data loggers, we discovered that the brain temperatures of the four wildebeest during that capture flight were the same as carotid arterial blood temperature (Fig. 1), and indeed have been among the highest organ temperatures that we ever have recorded (Jessen *et al.*, 1994). They were without consequence for the wildebeest, which went back into the herd and produced offspring in the next breeding season.

Those four data points demonstrated that the capacity for selective brain cooling, the process by which heat exchange in the carotid rete lowers brain temperature below arterial blood temperature in artiodactyls, felids and canids, was abandoned when the wildebeest seemingly most needed to cool their brains. Why was that important for conservation physiology? Because if selective brain cooling is not there to protect the brain against overheating, what does it do? What it does is to modulate the temperature of the thermoregulatory circuits in the hypothalamus that control evaporative cooling, allowing mammals with a rete to switch evaporative cooling on and off. When they can, they reduce evaporative cooling and increase non-evaporative cooling, so saving body water. When they need evaporative cooling, for example in fight and flight, they can switch it on instantaneously. The ability to save body water so as to use it for purposes other than cooling is a major benefit for any arid-zone animal, and it may be life-saving for the large mammals of the southern hemisphere, for which the main threat, under climate change, is aridity not heat. That is an idea my research team continues to pursue today (Strauss *et al.*, 2017).

My research career had ventured into the physiology of aridity long before it ventured into selective brain cooling, or even into non-human mammals, and the jumping-off point was an idea in conservation pathophysiology. Arguments about the benefits and costs of fever have been waging since ancient Greece, if not earlier. If it is beneficial, should we treat it? The pendulum swung massively towards benefits when another physiology legend of the 20th century, Matt Kluger (then University of Michigan, USA), pushed back the evolutionary origin of fever first to lizards and then much further (Kluger, 1979). If it had such an ancient evolutionary origin, surely it must benefit the febrile animal? My late colleague Helen Laburn, with whom I worked from 1972 until she passed away prematurely in 2014, and I had an ongoing programme of research in fever pathophysiology, and, in research already some way from gold miners, we could not reproduce Matt’s results in African lizards, or snakes, or tortoises. Then we noticed that all the lizard species in which Matt had demonstrated fever and its benefits were iguanids. Iguanids are peculiar lizards, and not just because most species choose to live in the Americas. They tend to sun-bask or otherwise choose environments which allow them attain mammal-like body temperatures. All of our African reptiles had much lower preferred body temperatures. Was there an African lizard species with a mammal-like preferred temperature? That was a question that we asked the late Gideon Louw, gentle and profoundly-knowledgeable Professor of Zoology at the University of Cape Town, who, more than anyone else, fuelled my enthusiasm for comparative physiology. Gideon told us about *Gerrhosaurus*(then *Angolosaurus*)*skoogi*, an elegant lizard of the Skeleton Coast, Namibia, where it lives on the hot dunes of the Namib Desert, emerging from a life in the sand to graze on the spiky cucurbit !nara (*Acanthosicyos horridus*). Gideon sent us to Mary Seely (then Director of the Gobabeb Research and Training Centre), still, in my view, the world’s leading desert biologist. Though *skoogi* had iguana-like body temperatures, it failed to develop fever, just like all other African lizard species (Mitchell *et al.*, 1990), but the excitement of that discovery was overwhelmed by my excitement about the Namib Desert, to which no prospective roadmap ever would have led me. The Namib is said to be the world’s oldest desert, and second in dryness only to the Atacama. It supports a wealth of animal life though, the physiology of which has kept me engaged in research, with Mary, for nearly forty years, with lizards still playing leading roles (Nagy *et al.*, 1991). The central Namib Desert sand sea now is a UNESCO World Heritage Site, supporting and desperately needing research in conservation physiology, a need made even more compelling because of the possible disappearance, under global warming, of the fog which sustains virtually all animal life there.

Although a prospective research roadmap would have told me that I could not go to two different destinations simultaneously, my retrospective map reveals many concurrent destinations. The research on selective brain cooling was a gateway into research on regulation of body temperature and of water balance in large terrestrial mammals, and not just African mammals. The Australian arid-zone macropods long ago adopted a water-saving lifestyle to which African mammals may have revert under climate change; they are nocturnally active. I have helped Shane Maloney (University of Western Australia) investigate the physiology of free-living western grey kangaroos *Macropus fuliginosus*, using biologgers (Maloney *et al.*, 2011), and learnt a lot, and not just about macropods, from the expert Terry Dawson (University of New South Wales). My veterinary surgeon and physiologist brother Graham Mitchell, who mastered the technique of implanting radiotemeters not just in large African mammals but in the foetuses of sheep and goats, which then delivered young with radiotelemeters implanted (Laburn *et al.*, 1992), decamped to the University of Wyoming, Laramie. I went to help investigate pronghorns *Antilocapra americana*, the ‘American antelopes’ that are not antelopes, and which endure a swing of ambient temperature (−30°C to +40°C) bigger than that endured by any African mammal (Lust *et al.*, 2007). We went to Saudi Arabia, where Arabian oryx *Oryx leucoryx* and sand gazelle *Gazella subgutturosa marica* live in a desert with temperatures already higher than any African desert will reach in the next century, even under the worst climate change scenarios. They have no surface drinking water for eleven months of the year. That was where our biologgers told us that homoeothermy, far from being a defining characteristic of mammals, is a luxury with which antelope dispense if maintaining homoeothermy compromises water and energy homoeostasis (Hetem *et al.*, 2016).

**Figure 2:**
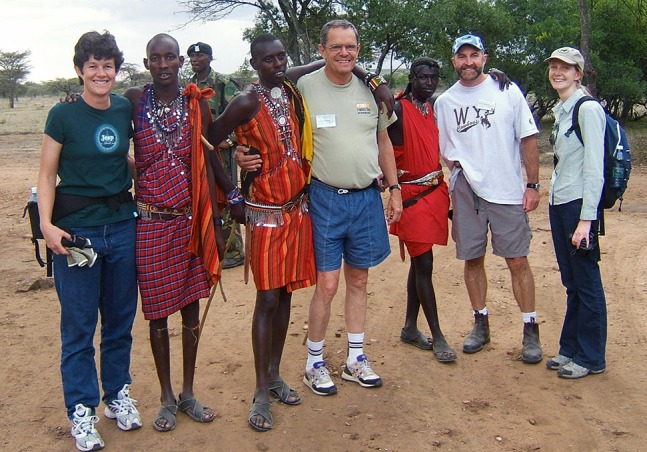
Andrea Fuller, Duncan Mitchell, Shane Maloney and Robyn Hetem (left to right), together with warriors, in the Maasai Mara, Kenya, 2008.

Who is ‘we’? Though it raises disapproving eyebrows in funding agencies, I regard as one of the greatest achievements of my career that less than 3% of the papers on which I have been an author have been single-author papers, and almost all of those were published before 1980. There are many rational arguments for conducting animal research, and especially large animal research, in teams, like the need for complimentary skills and for safety in the field. But those are not the compelling reasons for me. Although there are brilliant solo musicians, I like the sound of the orchestra, and I like playing in a research orchestra. On my papers that could qualify as conservation physiology and published in the 21st century, the recurring author names are Andrea Fuller, Shane Maloney, Robyn Hetem (Fig. 2), and Leith Meyer. All were former PhD students or postdoctoral fellows; all now are principal investigators in conservation physiology. Together we have discovered that the potentially catastrophic hyperthermia exhibited by antelope during capture is a fright response not a flight response, that we can reduce respiratory depression in opioid-darted mammals, that cheetah abandon hunts but not because they overheat, that dehydration drives selective brain cooling, that vervet monkeys cope better with thermal stress if they have friends, that elephants can maintain homoeothermy at ambient temperatures above body temperature in spite of not sweating and that aardvarks succumb in hot dry Kalahari summers not because they are heat intolerant but because their termite and ant prey are.

How would I advise a researcher aspiring to construct and lead a team working in vertebrate conservation physiology, as I have had the privilege to do? May I suggest instilling one value, teaching one skill, and generating one emotion? The value is care for the animals, from which will flow not just their ethical handling and the establishment of a relationship which reduces their stress, so getting their physiology as close as possible to normal, but a willingness to work in the early hours of the morning, which is when our sheep contrived to deliver their lambs. The skill is that of scientific writing, so that your team can communicate its research powerfully. I have encountered great researchers who speak badly, but never a great researcher who writes badly. And it is a skill, learnt by practice and by coaching, not a talent. The emotion is enthusiasm.
